# Microcystic adnexal carcinoma: report of rare cases

**DOI:** 10.1042/BSR20191557

**Published:** 2020-01-21

**Authors:** Lei Zhang, Xiaofeng Huang, Ting Zhou, Hongbao Cao

**Affiliations:** 1 Department of Oral Pathology, Nanjing Stomatological Hospital, Medical School of Nanjing University, Nanjing 210008, Jiangsu Province, China; 2 Department of Oral-maxillofacial Surgery, Nanjing Stomatological Hospital, Medical School of Nanjing University, Nanjing 210008, Jiangsu Province, China; 3 Department of Psychiatry, First Clinical Medical College/First Hospital of Shanxi Medical University, Taiyuan 030001, Shanxi Province, China; 4 School of Systems Biology, George Mason University, Fairfax, VA 22030, U.S.A.

**Keywords:** differential diagnosis, Histopathology, Immunology, Microcystic adnexal carcinoma, salivary gland

## Abstract

Microcystic adnexal carcinoma (MAC) is a rare, locally aggressive malignant neoplasm that derives from cutaneous eccrine/apocrine glands. MAC is classified as an eccrine/apocrine gland tumor and usually occurs in the skin. Here, we characterized and compared two cases of MAC. One is extremely rare in terms of its occurrence in the tongue. The other occurred in the lip, which is common. Histories of disease, diagnosis, and differentials were reviewed by the attending physicians. Hematoxylin and Eosin (HE) slides were evaluated by an experienced pathologist. Immunological markers for malignant eccrine/apocrine gland tumors were used to characterize the tumor’s nature. The examined markers included EMA, CK5/6, CK8/18, CK7, CK20, p63, S-100, Calponin, CD10, MYB, Bcl-2, Her-2, CD34, SMA, p53, CD43, CD117, and Ki-67. Both patients were males, presented with painless lumps in the lower lip and in the tongue, respectively. Both lumps were similar in terms of appearance, being whitish, and infiltrative with irregular borders. Both tumors also had similar histological features with nests of bland keratinocytes, cords, and ductal differentiation filled with Periodic acid–Schiff (PAS)-positive eosinophilic material. In both cases, circular or ovary tumor cells invaded into muscles and nerves. All tumor cells were CK5/6, CK8/18, EMA, and CK7 positive. Particularly, keratinocytes were p63 positive, and paraductal cells were p63, S-100, and SMA positive. Therefore, the rare case of MAC in the tongue appears to derive from the salivary gland.

## Introduction

Microcystic adnexal carcinoma (MAC) was first described by Goldstein et al. in 1982 [1] as a cutaneous malignancy originating from pluripotential adnexal keratinocyte. Since then, approximately 300 cases have been reported, and MAC is now typically described as a low-grade eccrine/apocrine gland cancer [2,3]. Though MAC is classified as low-grade tumor and rarely metastasizes to distant organs, it is locally aggressive, usually infiltrates deeply, and can penetrate into muscle, cartilage, bone, and nerves [4,5]. MAC is characterized by pilar and eccrine biphasic differentiation and sclerosing stroma [6]. The gross microscopic appearance usually comprises keratin cysts, nests, and cords of bland keratinocytes, and well-differentiated ducts [7]. Furthermore, numerous immunological markers have been reported to be positive in MAC, including CK5/6, CK8/18, EMA, and CK7 [8,9]. However, none by itself is sensitive and specific enough for the diagnosis of MAC.

In general, MAC originates from the adnexal glands in the skin. The most common sites involved are the face and lips [10]. Other sites, such as scalp [11], axilla [8,12], and trunk [13] can also be involved. Interestingly, MAC rarely occurs on the tongue or other oral mucosal tissues [14,15]. So far, no involvement of the anterior–ventral part of the tongue has been reported. Here, we characterized the clinical course, histopathological, and immunological features of the first case of MAC that occurred on the anterior–ventral surface of the tongue. Our results are valuable additions to the diagnosis and pathogenesis of MAC.

## Methods

Histories of illness, diagnosis, differentials, and Hematoxylin and Eosin (HE) slides were reviewed by the attending physicians. The demographic and clinical characteristics of these two patients were collected, as shown in Table 1. Fresh tissues were fixed in formaldehyde and embedded in paraffin wax. Serial sections of 4 μm were cut followed by HE staining. Sections were then used for immunostaining with corresponding antibodies. Specifically, immunological markers for malignant eccrine/apocrine gland tumors were used to characterize the tumor’s nature. These markers were EMA, CK5/6, CK8/18, CK7, CK20, p63, S-100, Calponin, CD10, MYB, Bcl-2, Her-2, CD34, SMA, p53, CD43, CD117, and Ki-67. Mouse anti-CK8/18 (MAB-0650), mouse anti-Calponin (MAB-0335), and Periodic acid–Schiff (PAS) (MST 8051) were purchased from Aomai New Technology, Shengzhen, China. Mouse anti-MYB (EP769Y) was purchased from Abcam, U.S.A. Mouse monoclonal anti-EMA (IR629), CK5/6 (IR780), CK7 (IR619), CK20 (IR777), p63 (IR662), S-100 (IR504), CD10 (IR648), GFAP (ZO334), Bcl-2 (IR614), Her-2 (SK001), CD34 (IR632), SMA (IR611), p53 (IR616), CD43 (IR636), CD117 (A4502), and Ki-67 (IR626) antibodies and the goat anti-mouse secondary antibody (K5007) were purchased from Dako, Denmark.

**Table 1:** Demographic and clinical characteristics of the two patients

	Case1	Case2
Gender	Male	Male
Age (year)	51	66
Hypertension status	No	Yes
Smoke status	No	Yes, 7 cigarettes/data
Family history of cancer	No	No
Disease position	Front of left tongue	Left lower lip
Clinical phenotypes (other)	Medium in texture, inactive and positive for tenderness. Free tongue movement and slight numbness	Hard texture, inactivity, no tenderness, no numbness etc.
Follow- p	38 months without recurrence and metastasis	14 months without recurrence and metastasis

## Results

### Case 1

A 51-year-old male patient presented with a painless mass on the anterior–ventral surface of the tongue. The mass was noticed by the patient 6 months ago without any erythema, warmth, and swelling. During the last 10 days, the patient noted that the mass was getting bigger, along with numbness and tenderness in the tongue. The examination revealed a 1.5 cm × 1.5 cm, circular mass with a smooth, shining surface. No abnormal pigmentation or ulceration was noticed. The mass was solid and moderately firm. Its boundaries could not be determined due to invasion into the vicinity. The tongue had a full range of movement. No submental or cervical lymphadenopathy was noticed. A biopsy from the left lobe of the mass was performed. The initial pathological results were inconclusive, and a malignant epithelial tumor remained the major differential diagnosis. An extended incisional biopsy and thorough pathological evaluation were then performed.

### Case 2

A 66-year-old male complained of excessive growth of lump in the left lower lip for 2 months. The lump occurred 10 years ago and was surgically removed. The pathological diagnosis was cutaneous adnexal neoplasm with follicle differentiation. The lump recurred 6 months after resection with a very low growth rate over the past 10 years. During the past 2 months, the patient noted excessive growth of the lump. The patient denied the change in the texture of the lump and associated erythema, warmth, and swelling. Physical examination revealed a firm, fixed mass in the lower lip. The boundaries of the mass were irregular and extended to gingival sulcus without noticeable adhesions. The face was asymmetric. No tenderness to palpitation around the ear and the temporomandibular joint or clicking and crepitation in temporomandibular joint was noticed. The open width of the mouth was approximately 3.7 cm. The size of bilateral parotid glands was within normal limits, and the ducts were patent with clear secretions. The differential diagnosis for this case was MAC, basal cell carcinoma (BCC), and squamous cell carcinoma.

### Managements

**Figure 1:**
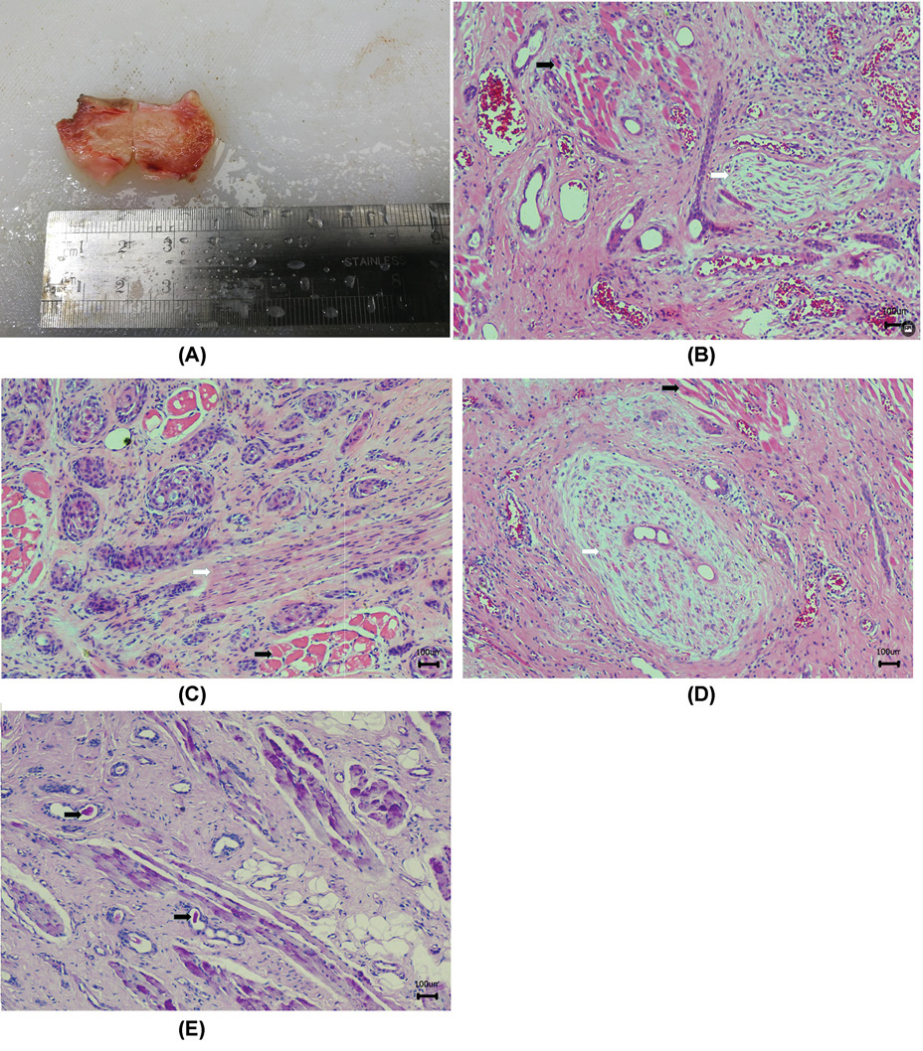
Histological features of case 1 and case 2 Macroexamination of Case 2 tumor mass (**A**) showed the tumor located between the skin and mucous membranes of the lips. The cut surface is gray and solid. HE staining of Case 1 (**B**) and Case 2 (**C,D**) showed tumor tissue is arranged in small glandular, nested or cord-like, showing skeletal muscle and nerve tissue invasion, interstitial collagen fibrosis. PAS staining of Case 1 (**E**) showed eosinophils in the gland. Black arrow indicates muscle, white arrow indicates nerve. The magnification is 100×.

In both cases, an extended resection was performed for further pathological evaluation. Regarding Case 1, the whole left lobe mass and submental and left submandibular lymph nodes were resected. The surface of the specimen of left lobe mass was yellow-grayish covered with mucosa, and the cut surface was grayish-white. The size of the mass was approximately 0.8 cm × 0.6 cm × 0.5 cm. The extended resection around the left lobe mass was a solid mass of tissue approximately 3.5 cm × 2.5 cm × 2.5 cm with surgical sutures in the mucosal surface (Case 2, see Figure 1A). The incision length was approximately 1.5 cm with grayish-red and grayish-white cut surfaces. All surgical specimens were fixed with 10% neutral formalin, embedded in paraffin, cut into 4-μm-thick serial sections, and stained with HE. Microscopic examination revealed the tumor tissue penetrated deeply, invading into the local fibers, striated muscles, and nerves (Case 1, see Figure 1B; Case 2, see Figure 1C,D). Histologically, numerous small-glandular squamous cell cords and nests were presented (Figure 1B,C). Well-differentiated ducts were also noted (Case 1, see Figure 1B; Case 2, see Figure 1D). Eosinophilic PAS-positive material was detected in the glandular lumens (Fuzhou Maixin Company, Fuzhou, China) (Figure 1E). The tumor cells were round or oval without nuclear atypical or mitotic figures. Hyperplasia and collagenization were noted in the local interstitial connective tissue. Tumor cells from five fields selected from different sections were counted manually. The cells with any signal intensity of Ki-67 staining was defined as Ki-67 positive. The ratio of Ki-67-positive cells to the total tumor cells observed were defined as Ki-67 proliferation index [16]. The Ki-67 proliferation index was less than 5% in the tissues. Noteworthy, the tumor tissue was mixed with some normal glands, which were identified by the serous acini (Figure 1B–D).

Immunohistochemical properties of tumor cells were determined by using the EnVision two-step method followed by diaminobenzidine (Dako, Denmark). All tumor cells were CK7, CK5/6, CK8/18, EMA positive (Table 2 and Figure 2A,B). However, the paraductal cells were p63, S-100, and SMA positive, and keratinocytes were p63 positive (Table 2 and Figure 2C,D). In the meantime, other markers, including CK20, CD43, CD117, Calponin, MYB, Bcl-2, Her-2, and p53 were tested and turned out to be negative (Table 2 and Supplementary Figures S1–S13).

**Figure 2:**
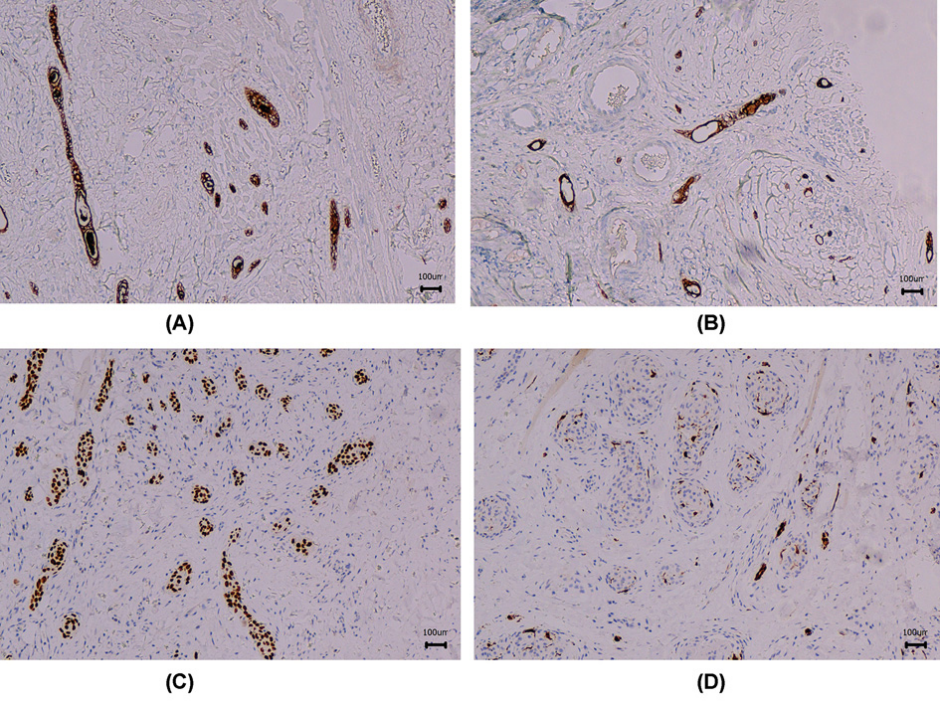
Immunohistochemical properties of tumor cells in Cases 1 and 2 The examples of anti-EMA (**A**), CK7 (**B**), P63 (**C**), and S100 (**D**). The magnification was 200×.

**Table 2:** The tumor immunohistochemical properties in Case 1 and Case 2

	CK5/6	CK8/18	EMA	CK7	CK20	p63	S-100	CD10	Calponin
Case1	+	+	+	+	-	+*	+**	+**	-
Case2	+	+	+	+	-	+*	+**	+	-

	MYB	Bcl-2	Her-2	SMA	p53	CD43	CD117	Ki-67	PAS
Case1	-	-	-	+*	-	-	-	*<5%+	+***
Case2	-	-	-	+*	+	-	-	*15%+	+***

+, Positive in tumor cells; −, negative in tumor cells; +*, positive in squamous cell carcinoma and paraductal cells adjacent to the tumor tissue; +**, positive in paraduct cells adjacent to the tumor tissue and positive in tumor cells; +***, positive in duct cell carcinoma and paraductal cells adjacent to the tumor tissue; +***, PAS positive in the ducts of tumor tissue. *Proliferative index.

Surgery is the most definitive management for MAC. In this case, wide local excision was performed 2.0 cm away from the edge of the tumor. Microscopic examination of tumor margins during the operation was used to ensure the tumor-free cutting margins. Incomplete incisions have been associated with high recurrence. Since MAC is a low-grade malignant gland cancer with low metastatic capacity, complete incisions are associated with much lower recurrence [17]. We followed up with this case for 14 months after the surgery, and no recurrence was observed. A long-term follow-up was scheduled, as 47% recurrence has been reported to occur within the first 3 years after resection [18]. Taken together, our results showed that, although Case 1 occurred in the tongue that is extremely rare, the Case 1 tumor shared the same histological properties with Case 2 that is far more common in terms of the anatomical region.

## Discussion

In these two cases, both patients presented with locally aggressive neoplasms with typical features of MAC. No predisposing factors such as radiation and excessive sunlight exposure were reported. In addition, the patient with MAC in the tongue had no habits of drinking hot beverages or areca chewing. More importantly, here we characterized and compared two cases of MAC, one is extremely rare and the other is more common in terms of its anatomical location.

MAC usually presents with eccrine/apocrine differentiation with ducts formed by squamous, basaloid keratinocytes, or epithelioid cells [19,20]. The hyperplasia cells aggregate into cords, strands, and sometimes nests that are surrounded by dense hyalinized stroma with thickened bundles [9,21]. Generally, the epidermal layer associated with MAC is normal. As MAC infiltrates deeply, tumor cells are usually found in the deep dermis or subcutaneous soft tissue with obscure boundaries. The adnexal structures are destroyed or diminished by the tumor [22]. MAC is locally aggressive with local perineural invasion as a characteristic feature [23]. In our two cases, the patient with a lesion in tongue showed perineural invasion, as evidenced by HE staining as well as symptoms of numbness and tingling of the tongue. In general, the tumor tissue displays a typical stratified structure from the superficial to the deep layer: small solid or cystic structures in the superficial portion, small ducts with fine structures in the middle portion, and interstitial sclerosis with tumor cells that have Indian fire-like arrangement in the deep portion. The tumor in the tongue is mainly located in the muscle layer of the tongue body without involving ventral and back mucosa. The histopathological configuration of this case includes small nest bucks formed by basal cells, small cyst or ductal structures that infiltrate vertically to the muscle layer. Microcysts contained PAS positive substance in their small ducts. The morphology of basal cell nest-like structures is very close to hair follicles.

Most MACs are derived from the cutaneous sweat glands. Lingual glands are an extremely rare source of MAC. Here, we reported one case of neoplasm in the tongue that had the typical histopathological features of MAC. To date, few cases of MAC in tongue have been reported. Based on their histological properties, lingual glands can be divided into three categories: anterior, posterior, and Ebner’s glands [24]. The anterior-lingual glands under the mucous membrane near the tip of the tongue are mixture of mucous and serous acini, while the posterior-lingual glands in the tongue’s base and margins and Ebner’s glands between the muscle fibers of the tongue in the lower half of the circumvallate papillae trench wall are pure mucous acini and serous acini, respectively. In our tongue MAC case, a mixture of acinus was presented around the tumor, suggesting the tumor might drive from the anterior-lingual glands. However, due to the anterior extension of Ebner’s gland in the tongue body [25], the tumor might be derived from Ebner’s gland as well. As the serous acinar of lingual glands and the sweat gland acinar of skin have similar histological structure and cellular construction, it is not a surprise that the MAC in the tongue has exactly the same pathologic features as the MAC derived from the sweat glands [6,15].

The morphology of MAC tumor cells is complex, presenting similarity with both basal cells and squamous cells. Generally, they are well differentiated and have a small cell size and rare mitotic figures [26]. In some cases, the tumor tissue has the features of sebaceous glands as indicated by a similar sheath area differentiation [19], suggesting the tumor cells can be differentiating toward the hair and sebaceous gland apocrine sweat unit. However, in some other cases, tumor tissues are completely composed of ducts. In such cases, these tumors are known as ‘syringomatous carcinoma’ or ‘sclerosing sweat duct carcinoma’, suggesting those tumor cells might be derived from small sweat ducts [27]. In terms of the origin of MAC, immunohistochemistry can be informative and help distinguish MAC from other neoplasms with highlights on eccrine/apocrine differentiation. Multiple immunological markers have been proposed to characterize the MAC, such as CK, CK7, EMA, Ber-EP4, and Bcl-2 [28]. Particularly, EMA and CK immunostaining have the most reliability. CK stains epithelial cells in MAC, while EMA is a strong marker for ductal structures. However, no single immunological marker has proven to be absolutely reliable in the diagnosis of MAC. Here, we immunostained multiple markers that have been stained in MAC and compared our results with the previous immunohistochemical results. In our cases, the immunohistochemistry showed that all the tumor cells were CK, CK7, EMA, Ber-EP4, and Bcl-2 positive. All these markers have been reported in multiple MAC cases. In addition, the peripheral cells of glandular tumor tissue were p63, α-SMA, and S-100 positive [29]. Previous studies have shown that all of these markers are present in the sweat gland tumors [30,31]. Interestingly, less than 25% of tumor cells were p53 positive and less than 5% Ki-67 positive, which might explain the low proliferation index in our cases [1]. In addition, markers that are less frequent in MAC, such as CK20, Her-2, and CD34 are negative in our cases [1,15]. Taken together, our results indicate that our cases present morphological, histological, and immunological features of MAC.

Four different diagnostic options were used in the present study, as described in detail as follows. The first diagnosis was to check whether a tumor belongs to hamartoma (Choristoma). The mixed hamartomas of the tongue often present as a cystic lesion. Microscopically, mixed hamartomas of the tongue are more heterogeneous, usually including gastric mucosa, neuroglial tissue, sebaceous glands, and other tissues combined with tumor-like hyperplasia. Choristomas of bones and cartilages are typical features of hamartoma. Although residual salivary glands are present in some cases, they are well-differentiated and even functionally intact. Hamartoma is benign and has clear-cut boundaries. Our cases lack all these features, which make the diagnosis of hamartoma unlikely [32].

The second diagnostic option was to check whether a tumor belongs to adenoid cystic carcinoma (ACC). ACC is the most common malignant epithelial tumor that originates from the oral and maxillofacial salivary glands. Similar to MAC, ACC has a latitude growth rate and can be locally aggressive with a propensity to invade peripheral nerves. Thus, pain is a common chief complaint in ACC patients. Histologically, tumor tissue usually displays sieve, tubular, or solid nested arrangements with sieve-like structures. The most common immunological tumor markers in ACC are MYB, CD43, and CD117 [33]. In our cases, the chief complaints were painless lumps. In addition, the microscopic examination lacks typical sieve-like structures. Furthermore, immunostaining shows that the tumor tissue is MYB, CD43, and CD117 negative. Therefore, ACC is less likely in our cases.

The third diagnostic option was to see whether a tumor is a pleomorphous adenocarcinoma (PMA). PMA mainly originates from small salivary gland tissue in the palate, cheek, retromolar trigone, upper lip, tongue base, or other locations, and is more common in middle-aged women. Similar to MAC, it infiltrates deeply and lacks nucleus atypia. However, unlike MAC, it presents with the consistency of cell morphology and high diversity regarding tissue structures, including small leafy, nipple or nipple cystic, sieve, cord, or small duct-like structures. Compared with MAC, the tumor cells are small, round, or spindle. In addition, tumor cells often surround and invade blood vessels or nerves in a swirl-like or target-ring pattern. Mucus-like or vitreous degeneration is visible in tumor stroma [34]. Our cases present with small-glandular, small-squamous cell cord-like, and/or nested differentiation, without other diverse tissue structures, which distinguishes it from PMA.

The last option in diagnosis was to test if a tumor belongs to BCC. The typical features of BCC are shiny or pearly appearance and peripheral palisade arrangement formed by small, homogeneous basophilic cells. Unlike MAC, differentiation of BCC tumor cells is poor. Although low molecular weight CK can be positive in BCC, EMA, and CEA are rarely positive [35]. In our cases, the tumors lacked the typical peripheral palisade structure and were EMA and CK8/18 positive, which distinguishes this case from BCC.

## Conclusion

Based on the clinical presentations and pathological results, a diagnosis of MAC was given to both cases at the agreement of the attending physicians and the pathologists. Our cases demonstrate that MAC can be derived from eccrine/apocrine glands outside the skin. Despite different anatomic origins, the MACs have the same clinical features.

## Supplementary Material

Supplementary Figures S1-S13

## Abbreviations

ACC: adenoid cystic carcinoma
BCC: basal cell carcinoma
CEA: Carcinoembryonic Antigen
CK: Cytokeratin
EMA: Epithelial Membrane Antigen
HE: Hematoxylin and Eosin
MAC: microcystic adnexal carcinoma
PAS: Periodic acid–Schiff
PMA: polymorphous adenocarcinoma
