# Supplementary figures and images for: Microcystic adnexal carcinoma: report of rare cases
Source: Biosci Rep. 2020 Jan 21;40(1):BSR20191557. doi: 10.1042/BSR20191557 (PMC6974419; doi:10.1042/BSR20191557)

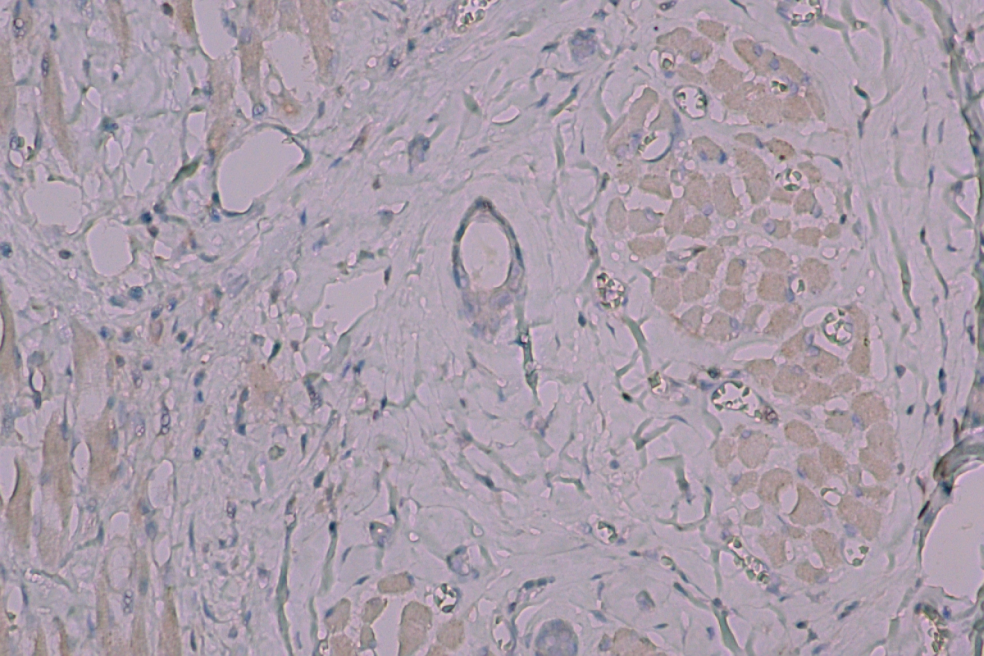

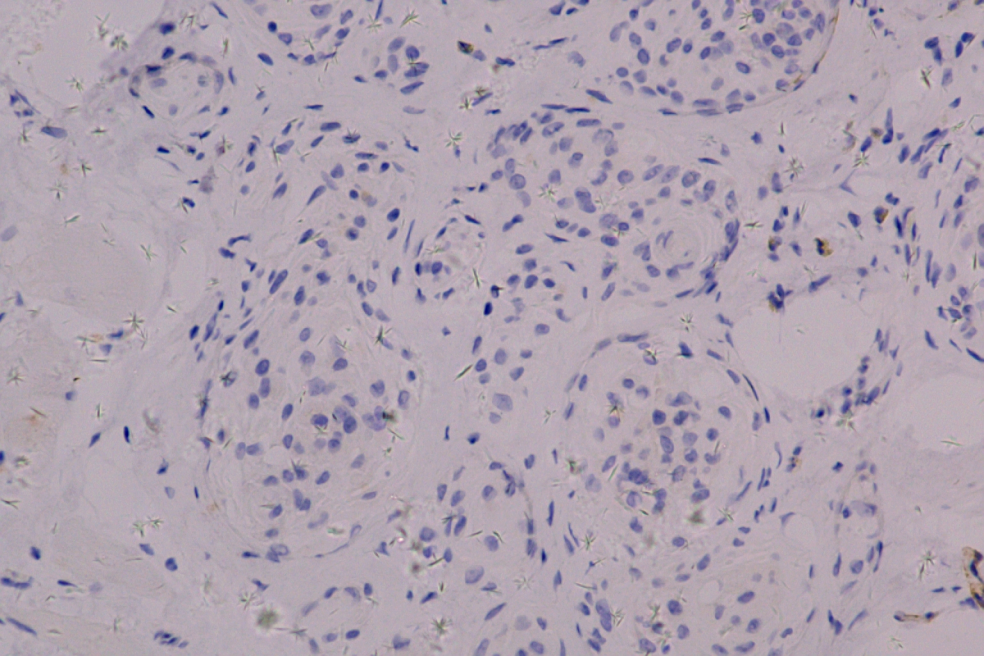

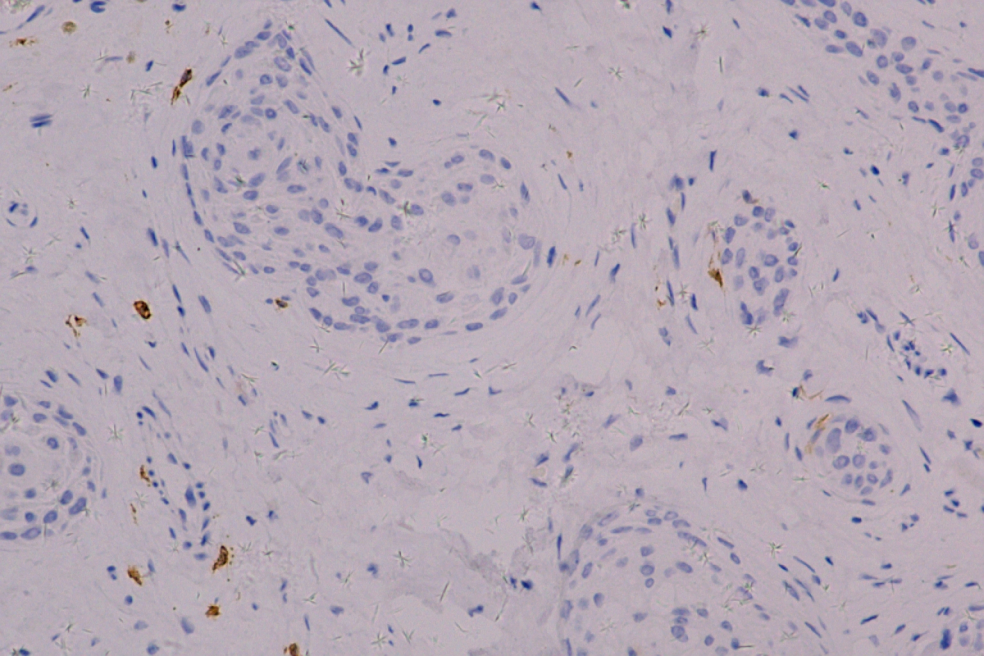

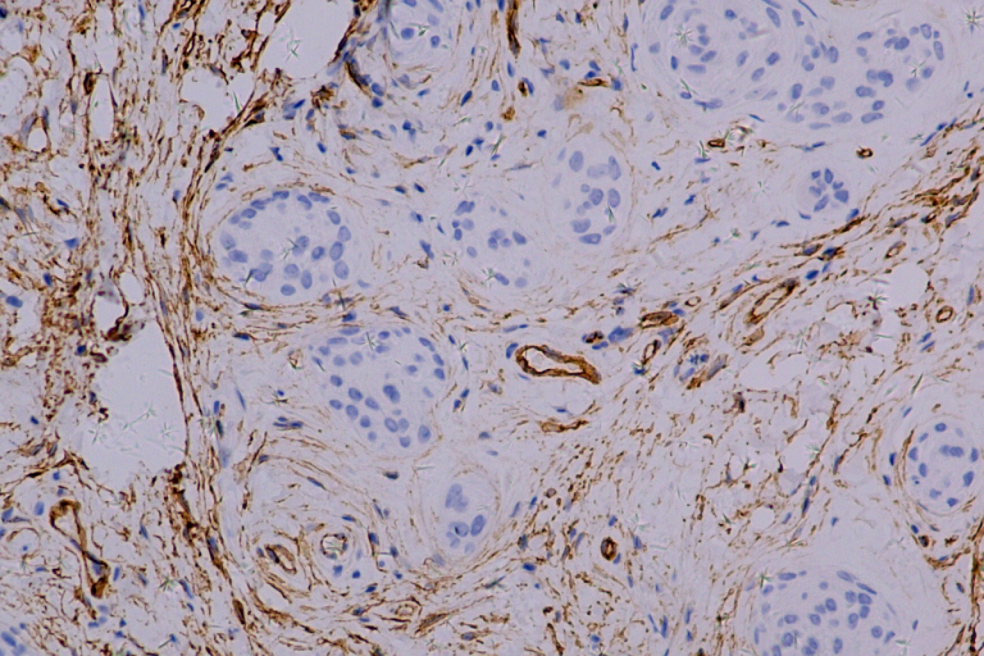

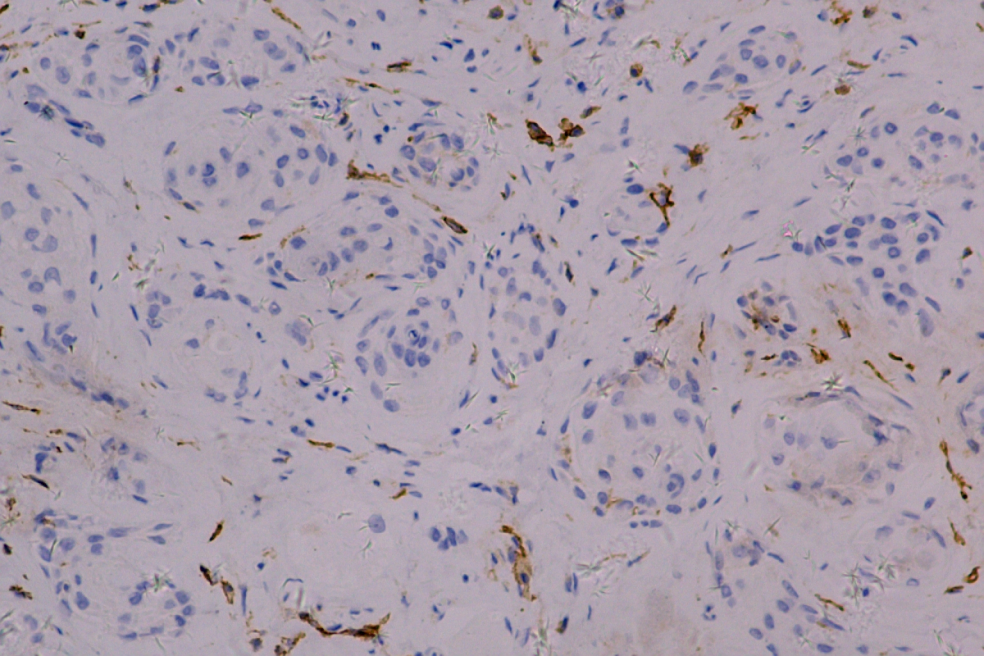

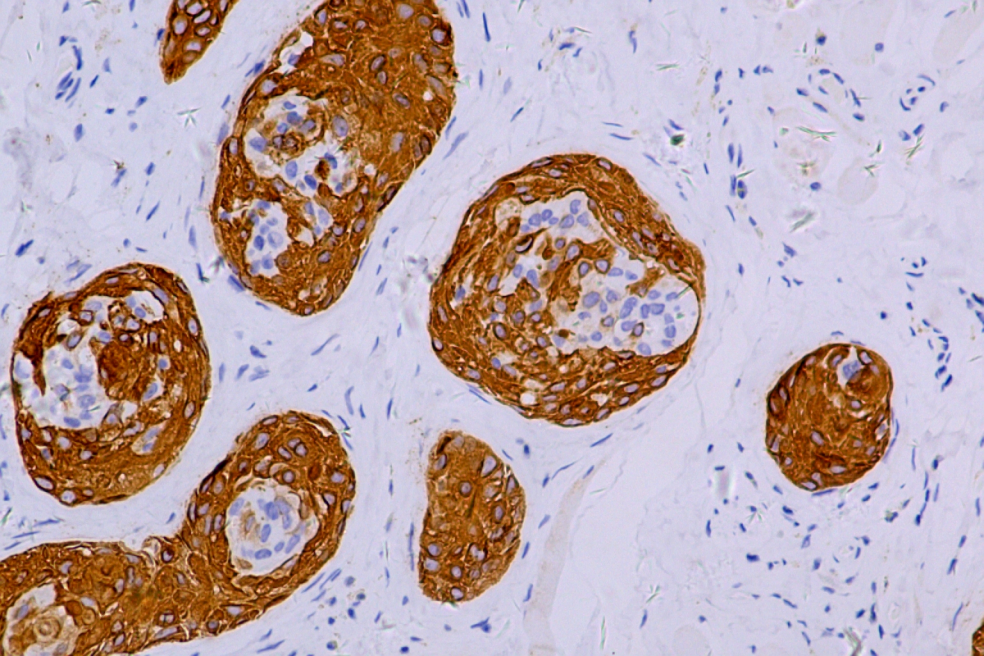

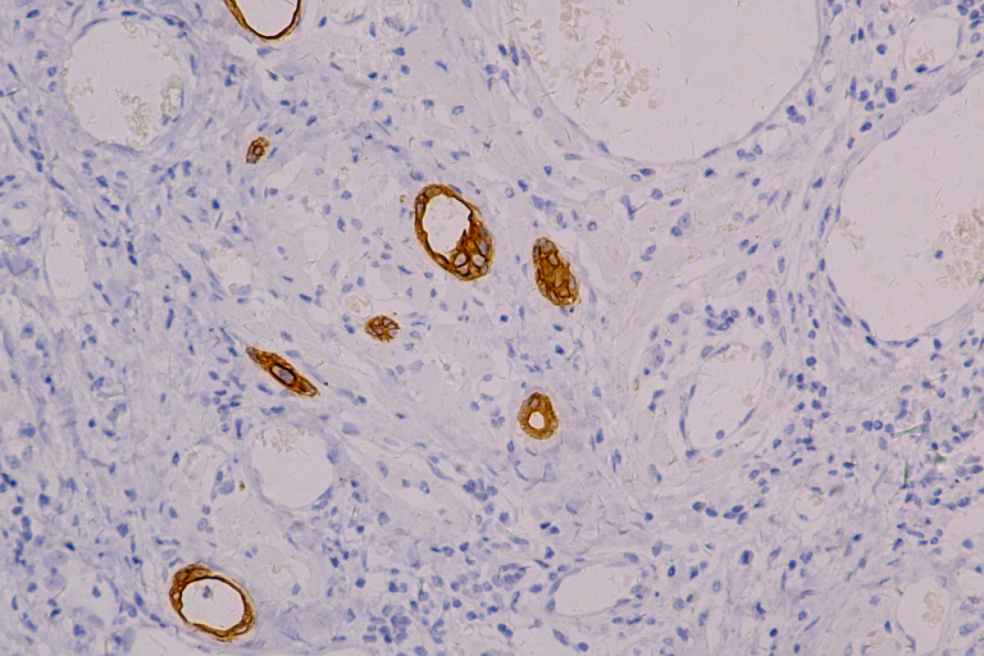

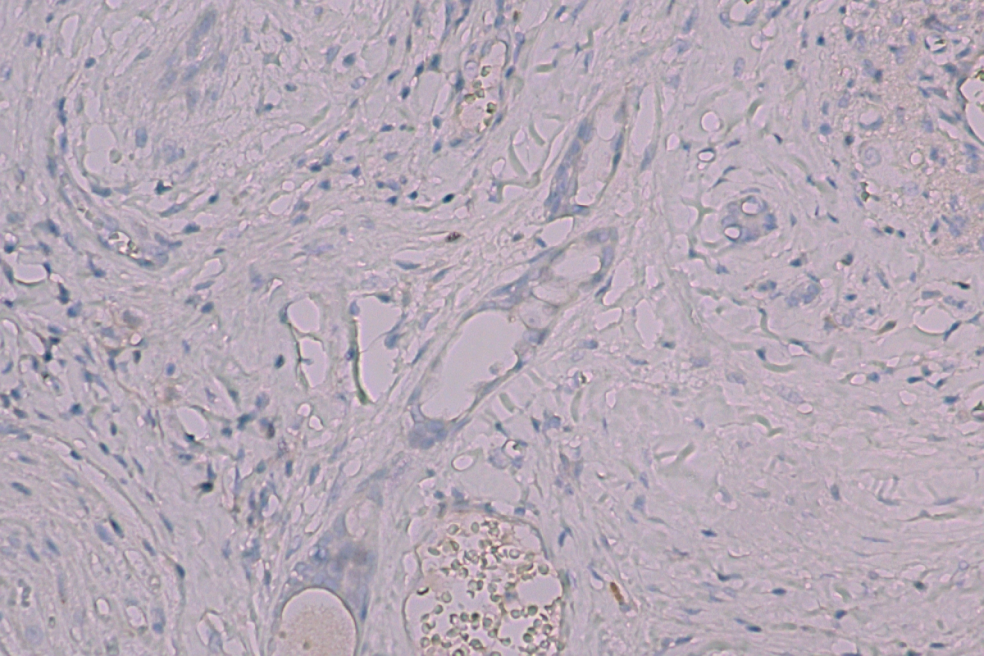

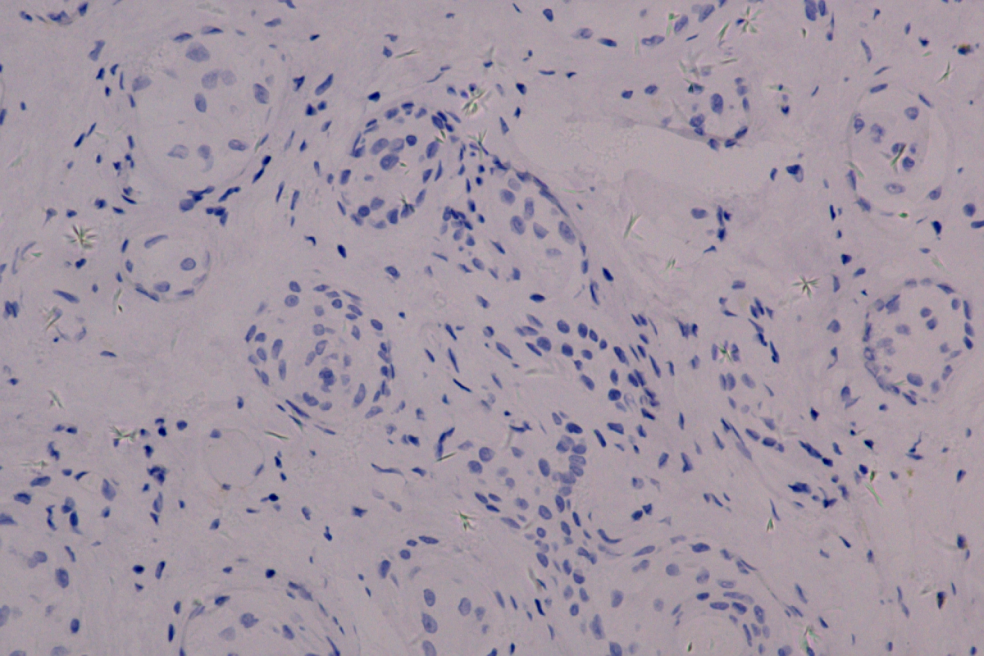

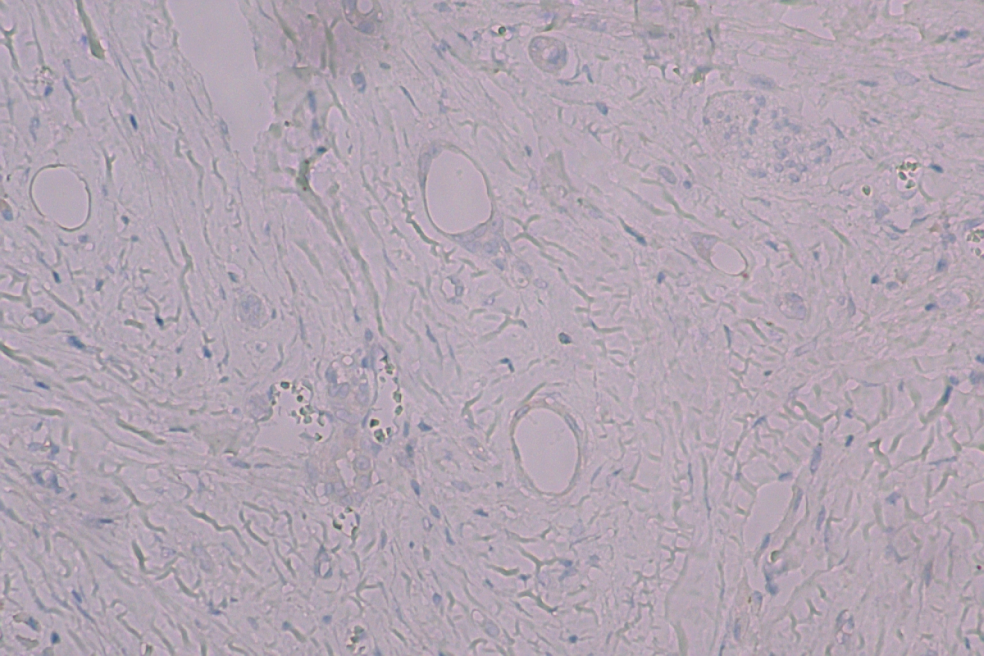

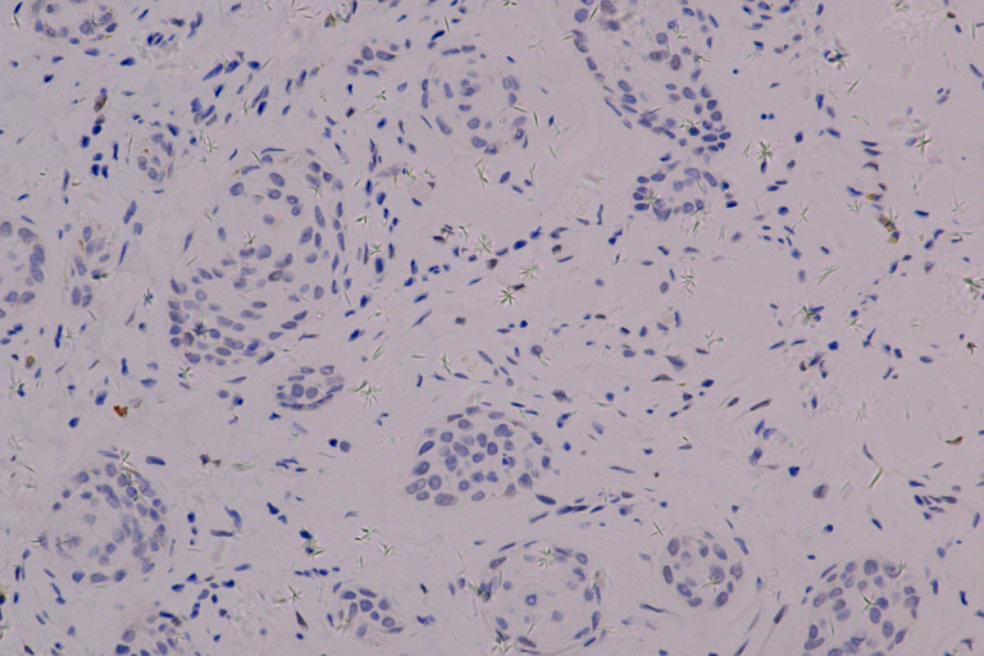

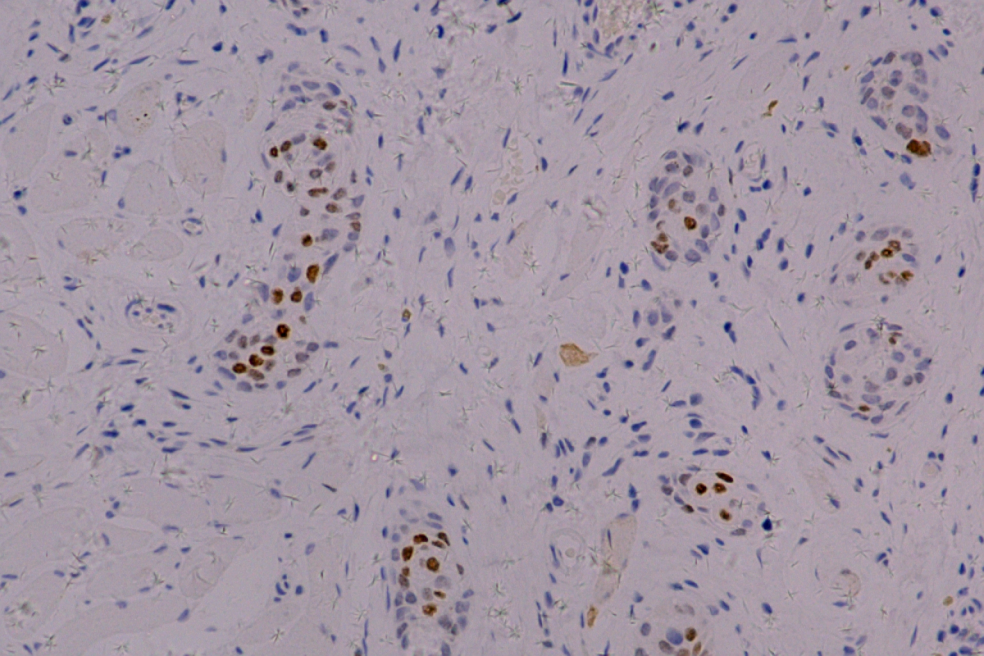

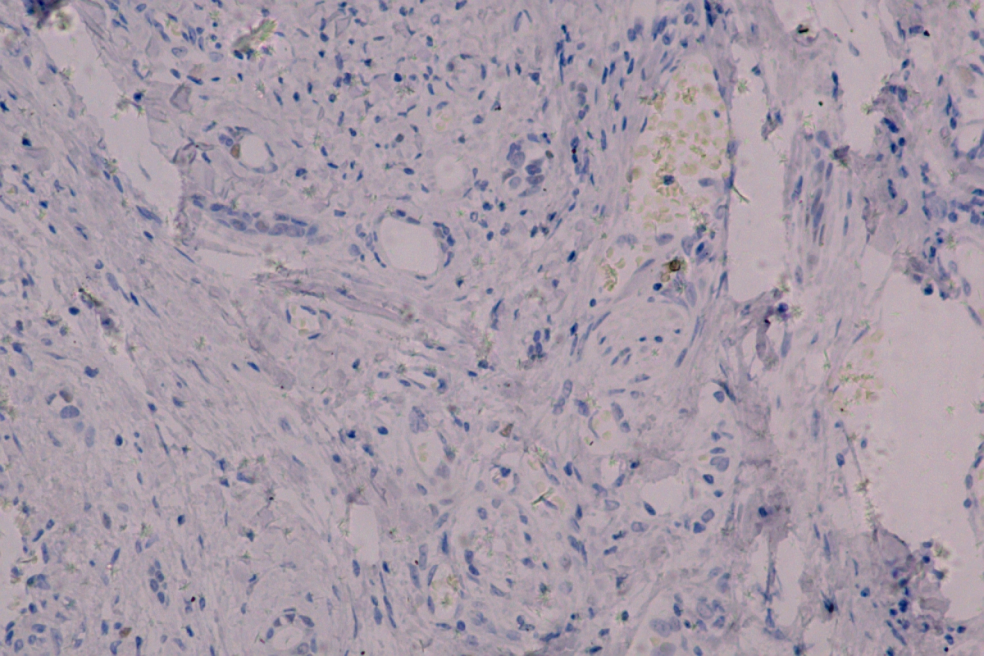

Supplement: Supplementary Figures S1-S13 [file BSR-2019-1557_supp.pdf]
